# Dacomitinib potentiates the efficacy of conventional chemotherapeutic agents via inhibiting the drug efflux function of ABCG2 *in vitro* and *in vivo*

**DOI:** 10.1186/s13046-018-0690-x

**Published:** 2018-02-20

**Authors:** Xiaoran Guo, Kenneth K. W. To, Zhen Chen, Xiaokun Wang, Jianye Zhang, Min Luo, Fang Wang, Shirong Yan, Liwu Fu

**Affiliations:** 1 State Key Laboratory of Oncology in South China, Guangdong Esophageal Cancer Institute, Sun Yat-Sen University Cancer Center, Guangzhou, 510060 China; 2 Hubei University of Medicine, Shiyan, Hubei 442000 China; 3 School of Pharmacy, Faculty of Medicine, the Chinese University of Hong Kong, Hong Kong, SAR China; 4 School of Pharmaceutical Sciences, Guangzhou Medical University, Guangzhou, 511436 China

**Keywords:** Dacomitinib, Multidrug resistance, ATP-binding cassette transporters, ABCG2

## Abstract

**Background:**

ATP-binding cassette subfamily G member 2 (ABCG2), a member of the ABC transporter superfamily proteins, mediates multidrug resistance (MDR) by transporting substrate anticancer drugs out of cancer cells and decreasing their intracellular accumulation. MDR is a major hurdle to successful chemotherapy. A logical approach to overcome MDR is to inhibit the transporter. However, no safe and effective MDR inhibitor has been approved in the clinic.

**Methods:**

The MTT assay was used to evaluate cell cytotoxicity and MDR reversal effect. Drug efflux and intracellular drug accumulation were measured by flow cytometry. The H460/MX20 cell xenograft model was established to evaluate the enhancement of anticancer efficacy of topotecan by dacomitinib *in vivo*. To ascertain the interaction of dacomitinib with the substrate binding sites of ABCG2, the competition of dacomitinib for photolabeling of ABCG2 with [^125^I]- iodoarylazidoprazosin (IAAP) was performed. Vanadate-sensitive ATPase activity of ABCG2 was measured in the presence of a range of different concentrations of dacomitinib to evaluate the effect of dacomitinib on ATP hydrolysis as the energy source of the transporter. A flow cytometry-based assay and western blotting were employed to study whether dacomitininb could inhibit the expression level of ABCG2. The mRNA expression levels of ABCG2 were analyzed by real-time quantitative RT-PCR. The protein expression level of AKT, ERK and their phosphorylations were detected by Western blotting.

**Results:**

Here, we found that dacomitinib, an irreversible pan-ErbB tyrosine kinase inhibitor (TKI) in phase III clinical trial, could enhance the efficacy of conventional chemotherapeutic agents specifically in ABCG2-overexpressing MDR cancer cells but not in the parental sensitive cells. Dacomitinib was found to significantly increase the accumulation of ABCG2 probe substrates [doxorubicin (DOX),Rhodamine 123 (Rho 123) and Hoechst 33342] by inhibiting the transporter efflux function. Moreover, dacomitinib stimulated ABCG2 ATPase activity and competed with [^125^I]-IAAP photolabeling of ABCG2 in a concentration-dependent manner. However, dacomitinib did not alter ABCG2 expression at protein and mRNA levels or inhibit ErbB downstream signaling of AKT and ERK. Importantly, dacomitinib significantly enhanced the efficacy of topotecan in ABCG2-overexpressing H460/MX20 cell xenografts in nude mice without incurring additional toxicity.

**Conclusions:**

These results suggest that dacomitinib reverses ABCG2-mediated MDR by inhibiting ABCG2 efflux function and increasing intracellular accumulation of anticancer agents. Our findings advocate further clinical investigation of combinations of dacomitinib and conventional chemotherapy in cancer patients with ABCG2-overexpressing MDR tumors.

## Background

ATP-binding cassette (ABC) transporters utilize the energy derived from ATP hydrolysis to actively pump out a wide variety of anticancer drugs from the inside of cancer cells, leading to reduced intracellular drug accumulation [1, 2]. Therefore, the overexpression of ABC transporters plays a critical role in multidrug resistance (MDR), which is the primary impediment to cancer chemotherapy, and it adversely affects the clinical outcome. To date, forty-nine human ABC transporters have been identified and they are divided into seven subfamilies (A-G) according to amino acid sequence similarities and phylogeny. Among these ABC transporters, the ATP-binding cassette subfamily B member 1 (ABCB1), the ATP-binding cassette subfamily C member 1 (ABCC1) and the ATP-binding cassette subfamily G member 2 (ABCG2), are considered the most relevant to MDR [3, 4].

Human ABCG2 was discovered in 1998 [5]. Overexpression of the ABCG2 transporter was observed in many tumor types such as breast cancer, colon cancer and melanoma [6–8]. The ABCG2 transporter can pump a wide variety of substrate drugs out of cancer cells, including most chemotherapeutic drugs such as mitoxantrone (MX), doxorubicin (DOX), topotecan, methotrexate, and irinotecan. Inhibition of ABCG2 is a logical approach to overcome MDR and it is considered clinically significant in cancer chemotherapy. Despite many compounds have been found to reverse ABCG2-mediated drug resistance *in vitro*, none of them has been approved for clinical practice because of insufficient efficacy, inherited side effects or unpredictable pharmacokinetic interactions [9–11]. Thus, developing novel inhibitors for MDR circumvention is urgent and important.

Tyrosine kinase inhibitors (TKI) are molecular targeted chemotherapeutic drugs that are highly promising for the specific treatment of a variety of cancers. They work by competing with ATP for the binding sites of the catalytic domain of oncogenic tyrosine kinases, thus inhibiting the downstream survival signaling of cancer cells. It is conceivable that TKIs also bind to ATP binding site of ABC transporter. In recent years, we have demonstrated that a number of TKIs, including lapatinib [12], alectinib [13] and apatinib [14], can be used to potentiate the efficacy of anticancer drugs in MDR cancer cells by inhibiting ABC transporters. The line of research suggested that TKIs may be developed into useful agents for circumventing MDR. Dacomitinib (PF299804) is an oral small molecule second-generation epidermal growth factor (EGFR) TKI under clinical development, which inhibits the tyrosine kinases of EGFR, ERBB2 and ERBB4. In a recent phase III study (ARCHER 1050 study), dacomitinib was found to produce a remarkable increase in progression free survival (PFS) of 5.5 months compared with gefitinib in the first line treatment of lung cancer patients with advanced EGFR-mutated non-small cell lung cancer [15]. Dacomitinib also exhibited potent anticancer activity in many cancer types *in vitro* [16–18]. In addition, the combination of dacomitinib with other targeted therapies have been reported to overcome acquired or de novo resistance to cancer chemotherapy [19]. In this paper, we evaluated the effect of dacomitinib on the reversal of ABCG2-mediated MDR.

## Methods

### Chemicals and Reagents

The 3-(4,5-Dimethylthiazol-yl)-2,5-diphenyltetrazolium bromide (MTT), dimethyl sulfoxide (DMSO), MX, topotecan, cisplatin (DDP), DOX, rhodamine123 (Rho 123), hoechst 33342, verapamil (VRP) and fumitremorgin C (FTC) were products of Sigma Aldrich Chemical Co (St. Louis, MO, USA). Dacomitinib (PF299804) and lapatinib were purchased from Selleck Chemicals (Houston, TX, USA). Dulbecco’s modified Eagle’s medium (DMEM), RPMI-1640 and fetal bovine serum (FBS) were products of Gibco BRL (Gaithersburg, MD, USA). Anti-total Akt, Anti-phospho-Akt, anti-total ERK1/2, anti-phospho-ERK1/2 antibodies and anti-ABCG2 antibodies were obtained from Santa Cruz Biotechnology (Paso Robles, CA, USA). The antibody against GAPDH was from Kangcheng (Shanghai, China). SYBR Green qPCR Master mix was purchased from ExCell Bio (Shanghai, China).

### Cell lines and Cell culture

The human non-small cell lung carcinoma cell line (NSCLC) H460 and its MX resistant ABCG2-overexpressing subline H460/MX20 [20]; the human oral epidermoid carcinoma cell line KB and its vincristine resistant ABCB1-overexpressing subline KBv200 [21]; the human colon carcinoma cell line S1 and its MX resistant ABCG2-overexpressing subline S1-MI-80 [22]; the human embryonic kidney cell line HEK293 and its stable HEK293/pcDNA3.1, wild-type ABCG2-482-R2 and mutant ABCG2-482-T7 cells were kind gift provided by Dr. Susan Bates (Columbia University/New York Presbyterian Hospital, Manhattan, NY, USA) [23, 24]. The cell lines were cultured in DMEM (H460, H460/MX20, S1 and S1-MI-80) or RPMI-1640 medium (KB and KBv200) supplemented with 10% fetal bovine serum at 37°C in a humidified incubator containing 5% CO_2_. All cells were grown in drug-free culture medium for more than two weeks before assay. Cell lines used in this study were thawed from early passage stocks and were passaged for less than 6 months.

### Cytotoxicity assays

The MTT assay was used to evaluate drug cytotoxicity *in vitro* [25]. Briefly, cells growing in logarithmic phase were harvested and resuspended in a final concentration of 2000~6000 cells/well, and seeded evenly in 96-well plates and incubated for 24 h at 37°C. To test the toxicity of dacomitinib, a range of different concentrations of dacomitinib were added into the wells. For the reversal experiments, different concentrations of conventional anticancer drugs were added into each wells after 1 h pre-incubation with dacomitinib, FTC or VRP. After 68 h of incubation, 20 μL of MTT solution (5 mg/ml) was added to each well. The MTT-medium was discarded and 150 μl DMSO was added to each well to dissolve the formazan crystals after 4 h. The absorbance was determined by a Model 550 microplate Reader (Bio-Rad, Hercules, CA, USA). IC_50_ (concentrations required to inhibit growth by 50% ) were calculated by the Bliss method. VRP, a specific ABCB1 inhibitor, was used as a positive control for KBV200 and KB cells [12]. FTC, a specific ABCG2 inhibitor, was used as the positive control for H460/MX20, H460, S1 , S1-MI-80, and ABCG2-transfected cells [26]. All experiments were repeated at least three times, and the mean values ± standard deviation (SD) were calculated.

### Animal experiments

The H460/MX20 cell xenograft model was established as previously described with minor modifications [27]. Athymic nude mice (BALB/c-nu/nu, female, 5 to 6 weeks old, 17~22 g) were provided by the Vital River (Beijing, China). H460/MX20 cells (3×10^6^) were subcutaneously injected into the right flank of athymic nude mice. When xenograft size reached 5 mm in diameter, the 28 mice were randomized into four groups receiving different treatments: (1) control (vehicle of dacomitinib, p.o., qod and saline i.p. qod); (2) dacomitinib (5 mg/kg, p.o., qod); (3) topotecan (2mg/kg, i.p., qod) and (4) topotecan (2mg/kg, i.p., qod) + dacomitinib (5 mg/kg, p.o., qod given 1 h before giving topotecan). The body weight and two perpendicular diameters (length and width) of the animals were measured every 2 days. Tumor volumes (V) were calculated according to the formula: V = (length × width^2^/2)^.^ The mice were euthanized when the mean of tumor weights reached 1 g in the control group. The xenografts were excised from the mice and weighed. The ratio of growth inhibition (IR) was estimated using the formula:

$$ IR\left(\%\right)=1-\frac{\mathrm{Mean}\ \mathrm{tumor}\ \mathrm{weigh}\ \mathrm{of}\ \mathrm{experiment}\ \mathrm{group}}{\mathrm{Mean}\ \mathrm{tumor}\ \mathrm{weigh}\ \mathrm{of}\ \mathrm{control}\ \mathrm{group}}\times 100\% $$

All animals received sterilized food and water. All experiments were approved by the Sun Yat-Sen University Institutional Animal Care and Use Committee.

### Intracellular drug accumulation assay

The intracellular accumulation assay of DOX, Rho 123 and Hoechst 33342 was measured by flow cytometry as previously described with minor modifications [28, 29]. Briefly, the logarithmically growing cells were seeded in six-well plates and incubated overnight at 37°C. The cells were incubated with 0.25,0.5, or 1 μM dacomitinib (or 2.5 μM FTC as the positive control) at 37°C for 3 h. Then 5 μM Rho 123,10 μM DOX, or 1 μM Hoechst 33342 were added to the medium and incubated for another 3 h or 0.5 h respectively. Finally, cells were washed with ice-cold PBS three times and resuspended in PBS for flow cytometry analysis.

### Rho 123 efflux assay

The effect of dacomitinib on the efflux of Rho 123 was tested in ABCG2-overexpressing cells as previous described [28]. S1 and S1-MI-80 cells were incubated with 5 μM Rho 123 for 0.5 h. Then the cells were collected, washed three times with cold PBS and subsequently incubated at 37°C with culture media with or without 1 μM dacomitinib. Subsequently cells were harvested at the designated time points (0, 15, 30, 60, and 120 min) and re-suspended in cold PBS buffer for flow cytometric analysis immediately.

### Detection of the cell surface expression of ABCG2

A flow cytometry-based assay was employed to study whether dacomitininb influenced the cellular localization of ABCG2 as described previously [28]. Briefly, H460/MX20 and S1-MI-80 cells were collected and washed three times with PBS containing 0.5% BSA, then treated with 1 μM dacomitinib at 4°C for 45 min. Finally, cells were washed twice with PBS buffer (supplemented with 0.5% BSA) and resuspended in 400 μL PBS buffer for flow cytometric analysis. Positive control samples were treated with FITC-conjugated anti-human Bcrp1/ABCG2 antibody in an identical manner, and negative control samples were incubated with FITC-labeled mouse immunoglobin G2b (IgG2b) antibody in parallel. All experiments were repeated at least three times.

### Photoaffinity labeling of ABCG2 with [125 I]-IAAP

Crude membrane from MCF7/FLV1000 cells overexpressing ABCG2 (50 μg protein) was incubated with 0 – 10 μM dacomitinib for 5 min at room temperature in 50 mM Tris-HCl (pH 7.5). Under subdued light, [^125^I]-IAAP (2200 Ci/nmole, 3 nM) was added and incubation was continued for another 5 min. The samples were then cross-linked by UV illumination (365 nm) on ice. BXP21 antibody was used to immunoprecipitate the labeled ABCG2. The samples were then subjected to SDS-PAGE using a 7% Tris-acetate NuPAGE gel, dried and exposed to Bio-Max MR film (Eastman Kodak Co., Rochester, NY) at -80^o^C overnight. Radioactivity incorporated into the transporter protein was quantified by using the Storm 860 PhosphorImager system (Molecular Dynamics, Sunnyvale, CA).

### ABCG2 ATPase assay

A colorimetric ATPase assay was carried out as described previously with some modifications [30]. Briefly, crude membranes isolated from MCF7/FLV1000 cells overexpressing ABCG2 (100 μg protein/mL) were incubated at 37°C with a range of different concentrations of dacomitinib in the presence or absence of 1.2 mM sodium orthovanadate in an assay buffer (50 mM KCl, 5 mM sodium azide, 2 mM EDTA, 10 mM MgCl_2_, 1 mM DTT, pH 6.8) for 5 min. ATP hydrolysis reaction was then started by the addition of 5 mM Mg-ATP (concentration in a final volume of 60 μL) and incubated for 10 min. SDS solution (30 μL of 10 % SDS) was then added to stop the reaction. After the addition of a detection reagent (35 mM ammonium molybdate, 15 mM zinc acetate, 10% ascorbic acid) and incubation at 37^o^C for 20 min, absorbance was subsequently measured at 750 nm. The amount of inorganic phosphate released was quantified by reading from a calibration curve. Specific dacomitinib-stimulated ABCG2 ATPase activity was determined as the difference between the amounts of inorganic phosphate released from ATP in the absence and presence of sodium orthovanadate.

### Western blot analysis

Protein expression was determined by Western blot analysis as previously described [31]. The protein expression level of ABCG2 was detected in H460/Mx20 and S1-MI-80 cells after treating with 0.25, 0.5 and 1 μM dacomitinib for 48 h. The protein expression level of AKT, ERK, and their phosphorylations were detected in H460/MX20 and S1-MI-80 cells and their parental cells.

### Real-time quantitative RT-PCR

mRNA expression level of ABCG2 was performed and analysed as described previously [32]. The total cellular RNA was isolated by Trizol Reagent RNA extraction kit (Molecular Research Center, Cincinnati, OH, USA) after cells were treated with different concentration of dacomitinib for 48 h. The PCR primers were as follows: 5′-TGGCTGTCATGGCTTCAGTA-3′ (forward) and 5′-GCCACGTGATTCTTCCACAA-3′ (reverse) for ABCG2, 5′-CTTTGGTATCGTGGAAGGA-3′ (forward) and 5′-CACCCTGTTGCTGTAGCC-3′ (reverse) for GAPDH. The relative expression of ABCG2 was quantified using the 2 –ΔΔCt method after normalization by GAPDH expression in each sample [33].

### Data Analysis

All data was presented as means ± SD. All experiments were repeated at least three times. The SPSS statistical software (SPSS 16.0) was used in Statistical analyses. Statistical differences were calculated using the Student’s *t* test and *p* < 0.05 was considered statistically significant. "*", *p*<0.05; "**", *p*<0.01.

## Results

### Dacomitinib increased the sensitivity of substrate anticancer drugs in ABCG2-overexpressing cells *in vitro*

**Fig. 1:**
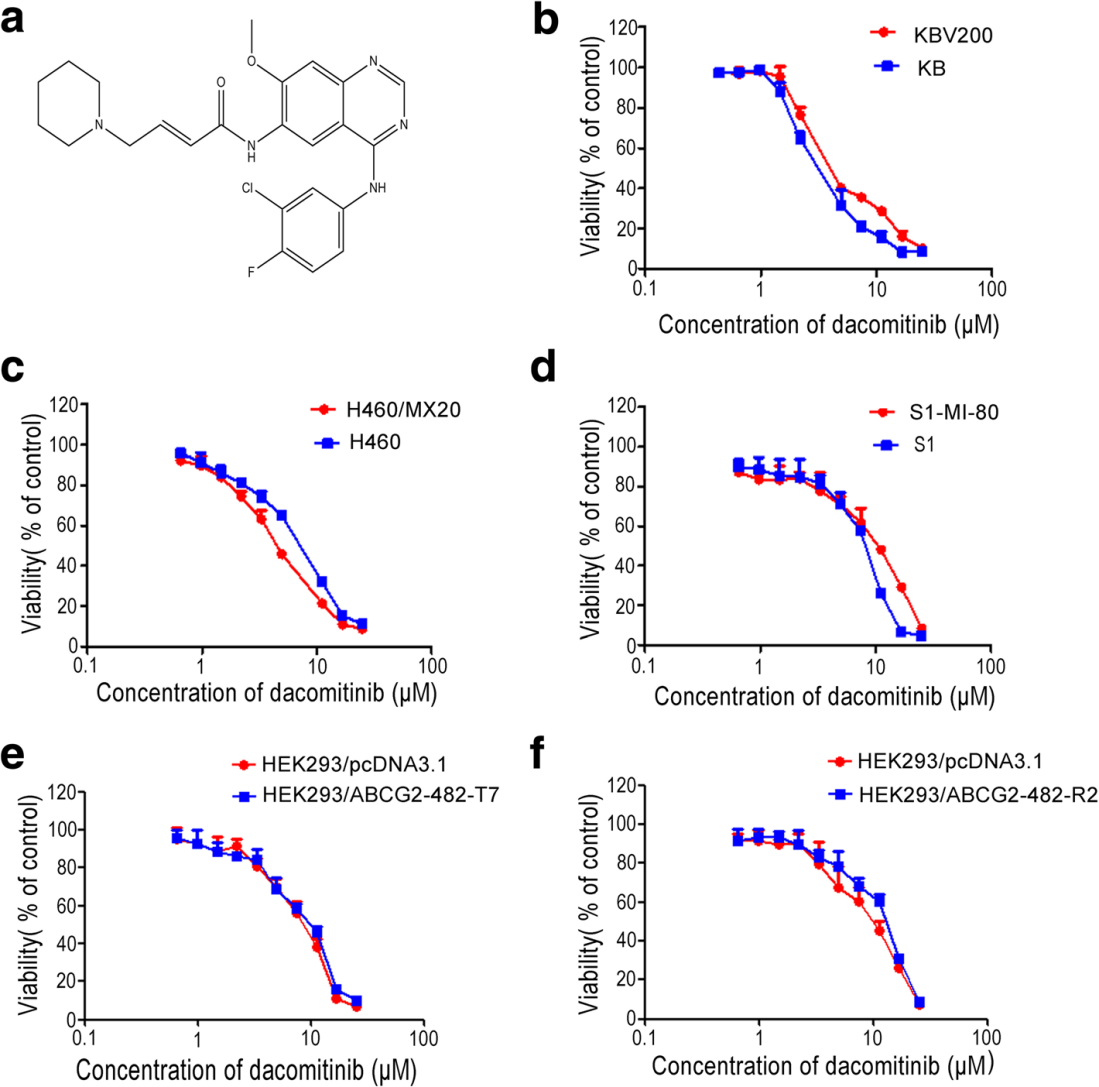
The structure of dacomitinib and cytotoxicity of dacomitinib. The structure of dacomitinib (**a**). MTT cytotoxicity assay was assessed in ABCG2 and ABCB1-over-expressing cells and their parental sensitive cells: ABCB1-negative KB and ABCB1-overexpressing KBV200 cells (**b**); ABCG2-negative H460 and ABCG2-overexpressing H460/MX20 cells (**c**); ABCG2-negative S1 and ABCG2-overexpressing S1-MI-80 cells (**d**); ABCB1-negative HEK293/pcDNA3.1 and ABCG2-overexpressing Wild-type ABCG2-482-T7 and (**e**); ABCG2-negative HEK293/pcDNA3.1 and ABCG2-overexpressing mutant ABCG2-482-R2 cells (**f**). Cells were treated with varying concentrations of dacomitinib for 72 h. Results from three independent experiments were expressed as the mean ± SD

The anticancer activity of dacomitinib, a few anticancer drugs and their combinations were evaluated in sensitive parental (KB, H460, S1) cell lines and their drug resistant sub-lines (KBV200, H460/MX20, S1-MI-80 ) by the MTT cell proliferation assay *in vitro*. There were more than 80% cells surviving with dacomitinib alone treatment at concentrations up to 1 μM (Fig. 1), so 0.25, 0.5, or 1 μM dacomitinib were tested in the drug combination experiments. While dacomitinib (1 μM) was found to significantly potentiate the anticancer activity of ABCG2 substrate anticancer drugs (MX and topotecan) in ABCG2 overexpressing H460/MX20 and S1-MI-80 cells, no notable potentiation effect was observed in the parental drug sensitive S1 cells and there was only minimal decrease of IC_50_ value of MX in H460 cells (Table 1). Dacomitinib did not alter IC_50_ value of DDP which was not an ABCG2 substrate in ABCG2 overexpressing S1-MI-80, H460/MX20 cells and their parental drug sensitive S1 and H460 cells (Table 1). The specific ABCG2 inhibitor (FTC, 2.5 μM) was used as a control for comparison of the resistance reversal effect.

**Table 1:** Effect of dacomitinib on enhancement of conventional chemotherapeutic agents

Compounds	IC_50_±SD (μM) (fold-reversal)	
	KB	KBV200(ABCB1)
DOX	0.0620±0.0030 (1.00)	0.4693±0.0299 (1.00)
+0.25μM Dacomitinb	0.0603±0.0114 (1.03)	0.3720±0.0176 (1.26)
+0.5μM Dacomitinb	0.0591±0.0121 (1.05)	0.3994±0.0865 (1.17)
+1μM Dacomitinb	0.0545±0.0102 (1.14)	0.2950±0.4790 (1.59)
+10μM VRP	0.0540±0.0141 (1.15)	0.0303±0.0180 (9.70)**
DDP	0.4477±0.0926 (1.00)	0.8727±0.0385 (1.00)
+1μM Dacomitinb	0.4958±0.0525 (0.90 )	0.9865±0.8774 (0.88)
	H460	H460/MX20(ABCG2)
MX	0.0254±0.0068 (1.00)	2.4569±0.0504 (1.00)
+0.25μM Dacomitinb	0.0309±0.0081 (0.82)	0.6534±0.0803 (3.76)**
+0.5μM Dacomitinb	0.0162±0.0104 (1.50)	0.1937±0.0105 (12.68)**
+1μM Dacomitinb	0.1529±0.0018 (1.12)	0.1892±0.0073 (12.98)**
+2.5μM FTC	0.1921±0.0011 (1.30)	0.3012±0.0233 (8.15)**
DDP	3.6240±0.8351 (1.00)	9.6831±2.3178 (1.00)
+1μM Dacomitinb	4.8224±0.9831 (0.75)	9.1475±1.8112 (1.05)
	S1	S1-MI-80 (ABCG2)
MX	0.1715±0.0262 (1.00)	8.5372±0.7831 (1.00)
+0.25μM Dacomitinb	0.1571±0.0144 (1.09)	1.6636±0.2819 (5.13)**
+0.5μM Dacomitinb	0.1369±0.0042 (1.25)	1.1503±0.0709 (7.42)**
+1μM Dacomitinb	0.1529±0.0018 (1.12)	0.6009±0.0128 (14.20)**
+2.5μM FTC	0.1405±0.0494 (1.22)	2.2289±0.3450 (3.83)**
Topotecan	0.7334±0.0785 (1.00)	83.1904±2.4666 (1.00)
+0.25μM Dacomitinb	0.9914±0.3053 (0.74)	72.2940±8.0594 (1.15)
+0.5μM Dacomitinb	0.5367±0.0921 (1.36)	15.9879±1.9511 (5.18)**
+1μM Dacomitinb	0.7060±0.0407 (1.03)	11.4639±1.0595 (7.24)**
+2.5μM FTC	0.8681±0.0611 (0.85)	7.7162±0.5868 (10.75)**
DDP	11.1384±1.6603 (1.00)	11.0324±0.1046 (1.00)
+1μM Dacomitinb	10.8398±1.0169 (1.02)	12.1685±0.7354 (0.90)

Cell viability was performed by MTT assay as described in “Materials and Methods”. VRP (specific inhibitor of ABCB1) and FTC (specific inhibitor of ABCG2) were used as the positive control. The fold reversal of MDR (values given in parentheses) was calculated by dividing the IC_50_ value for cells with the anticancer agent in the absence of dacomitinib by that obtained in the presence of dacomitinib. Data were shown as the mean ± SD of at least three independent experiments performed in triplicate. * *p* < 0.05, ** *p* < 0.01

It has been reported that mutations at amino acid 482 in ABCG2 affected the substrate specificity and undermine the effectiveness of ABCG2 inhibitor [24]. So, we investigated whether dacomitinib could potentiate the cytotoxicity of ABCG2 substrate drugs in cells transfected with either the wild-type (482R2) or mutant (482T7) forms of ABCG2. As shown in Table 2, the IC_50_ values for MX in ABCG2 transfected cell lines ABCG2-482-R2 and ABCG2-482-T7 cells were significantly higher than those in parental cell line HEK293/pcDNA3.1 cells, and dacomitinib significantly reduced the IC_50_ value for MX at 0.25 μM, 0.5 μM and 1 μM. It suggested that dacomitinib could reverse resistance to MX in cells expressing either wild-type or mutant ABCG2 (Table 2). There was no significant difference in the IC_50_ values for MX in HEK293/pcDNA3.1 cells with or without dacomitinib (Table 2). In addition, the anticancer activity of DDP (non-ABCG2 substrate) was not altered in any of the cell lines with or without the concomitant treatment of dacomitinib. These results showed that dacomitinib specifically enhanced the efficacy of ABCG2 substrate chemotherapeutic agents in ABCG2-overexpressing cells *in vitro*.

**Table 2:** Effect of dacomitinib on reversing and ABCG2-mediated MDR in stable-transfected cells

Compounds	IC_50_±SD (μM) (fold-reversal)		
	HEK293/pcDNA3.1	ABCG2-482-R2	ABCG2-482-T7
MX	0.0093±0.0012 (1.00)	0.0762±0.0020 (1.00)	0.0356±0.0266 (1.00)
+0.25μM Dacomitinib	0.0072±0.0004 (1.29)	0.0340±0.0026 (2.22)**	0.0171±0.0005 (2.08)**
+0.5μM Dacomitinib	0.0097±0.0003 (0.95)	0.0230±0.0011 (3.27)**	0.0098±0.0009 (3.63)**
+1μM Dacomitinib	0.0074±0.0001 (1.25)	0.0150±0.0021 (5.08)**	0.0092±0.0008 (3.86)**
+2.5μM FTC	0.0106±0.0016 (0.87)	0.0060±0.0013 (12.09)**	0.0025±0.0029 (12.40)**
DDP	2.5420±0.1392 (1.00)	1.0719±0.1109 (1.00)	0.8525±0.0502 (1.00)
+1μM Dacomitinb	2.2230±0.0433 (1.14)	0.9194±0.0177 (1.16)	0.9150±0.1908 (0.93)

Cell survival was performed by MTT assay as described in “Materials and Methods”. VRP (specific inhibitor of ABCB1) and FTC (specific inhibitor of ABCG2) were used as the positive control. The fold reversal of MDR (values given in parentheses) was calculated by dividing the IC_50_ value for cells with the anticancer agent in the absence of dacomitinib by that obtained in the presence of dacomitinib. Data were shown as the mean ± SD of at least three independent experiments performed in triplicate. *p < 0.05, **p < 0.01

### Dacomitinib significantly enhanced therapeutic effect of topotecan in H460/MX20 cell xenografts *in vivo*

**Fig. 2:**
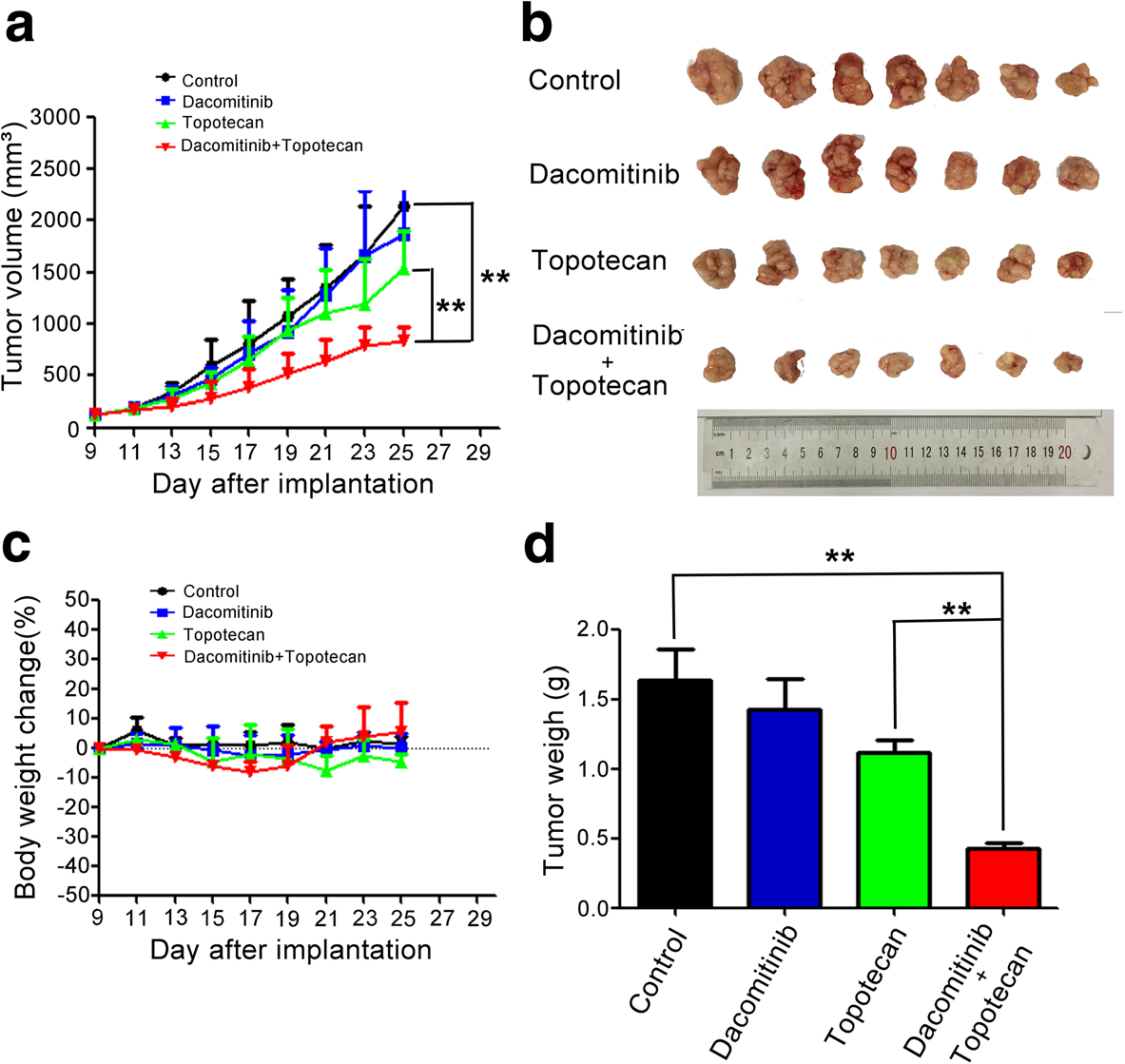
Dacomitinib enhanced the anticancer effect of topotecan in the H460/MX20 cell xenograft model in nude mice. The changes in tumor volume over time after the H460/MX20 cell implantation (*n* =7) (**a**). Data shown are mean ± SD of tumor volumes for each group. The image of tumors size in four groups excised from the mice on the 25th day after implantation (**b**). Average percentage change in body weight after treatments (**c**). Mean tumor weight after excising from the mice on the 25th day after implantation (*n* = 7) (**d**). The four treatment groups were: (1) control (vehicle of dacomitinib, p.o., qod and saline i.p. qod); (2) dacomitinib (5 mg/kg, p.o., qod); (3) topotecan (2mg/kg, i.p., qod) and (4) topotecan (2mg/kg, i.p., qod) + dacomitinib (5 mg/kg, p.o., qod given 1 h before giving topotecan). Results were presented as the mean ± SD. * *p*< 0.05, ** *p*< 0.01

To verify whether dacomitinib could reverse the resistance to conventional anti-cancer drugs *in vivo*, an ABCG2-overexpressing multidrug-resistant H460/MX20 cell xenograft model in nude mice were established. There were no significants differences in size and weight of tumor between animals treated with saline,dacomitinib or topotecan alone (Fig. 2 b,d). However, we found that the combination of dacomitinib and topotecan produced a significant greater inhibition of tumor growth in the group compared to other groups (*p* < 0.05). These results indicated that the antitumor efficacy of topotecan was significantly enhanced when combined with dacomitinib in ABCG2-overexpressing cell xenografts *in vivo*. Moreover, no appreciable weight loss or mortality were observed in the athymic nude mice, suggesting that the combination regimen did not cause additional toxicity (Fig. 2c).

### Dacomitinib augmented the intracellular accumulation of DOX and Rho 123 in MDR cells overexpressing ABCG2

**Fig. 3:**
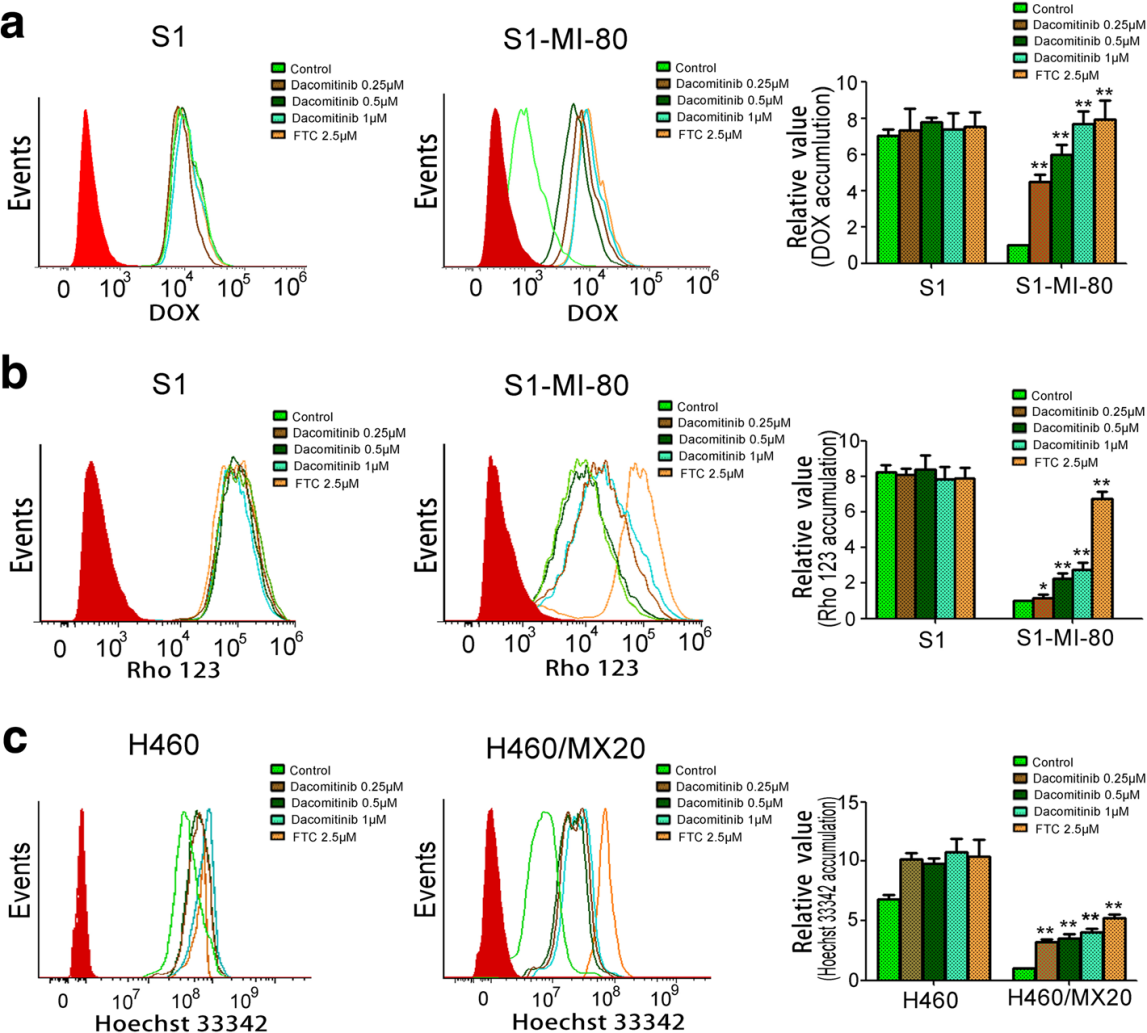
Effect of dacomitinib on the intracellular accumulation of DOX, Rho 123 and Hoechst 33342 in MDR cells and their parental drug sensitive cells. The accumulation of Rho 123 (**a**), DOX (**b**) in S1 and S1-MI-80 cells and Hoechst 33342 (**c**) in H460/MX20 cells were measured by flow cytometric analysis as described in “Material and Methods” section. The results were presented as fold change in fluorescence intensity relative to control MDR cells. Columns, means of triplicate determinations; bars means SD. * *p*< 0.05, ** *p*< 0.01

The data above revealed that dacomitinib significantly increased the sensitivity of MDR cancer cells with overexpressing ABCG2 to conventional substrate chemotherapeutic agents of ABCG2 *in vitro* and *in vivo.* As shown in Fig. 3, the intracellular accumulation of ABCG2 substrates DOX or Rho 123 in the resistant S1-MI-80 cells were remarkably lower than that in the parental S1 cells. This observation is consistent with the greater drug efflux function in the ABCG2-overexpressing resistant cells. Importantly, treating with 1 μM dacomitinib significantly increased the intracellular accumulation of DOX and Rho 123 in a concentration-dependent manner in S1-MI-80, but no significant difference in the parental sensitive S1 cells. Furthermore, similar results were showed where dacomitinib increased intracellular accumulation of ABCG2 substrate Hoechst 33342 in H460/MX20 cells. The data suggested that dacomitinib could increase intracellular accumulation of ABCG2 substrates in ABCG2-overexpressing cells.

### Dacomitinib decreased the efflux of Rho 123 in ABCG2-overexpressing cells

**Fig. 4:**
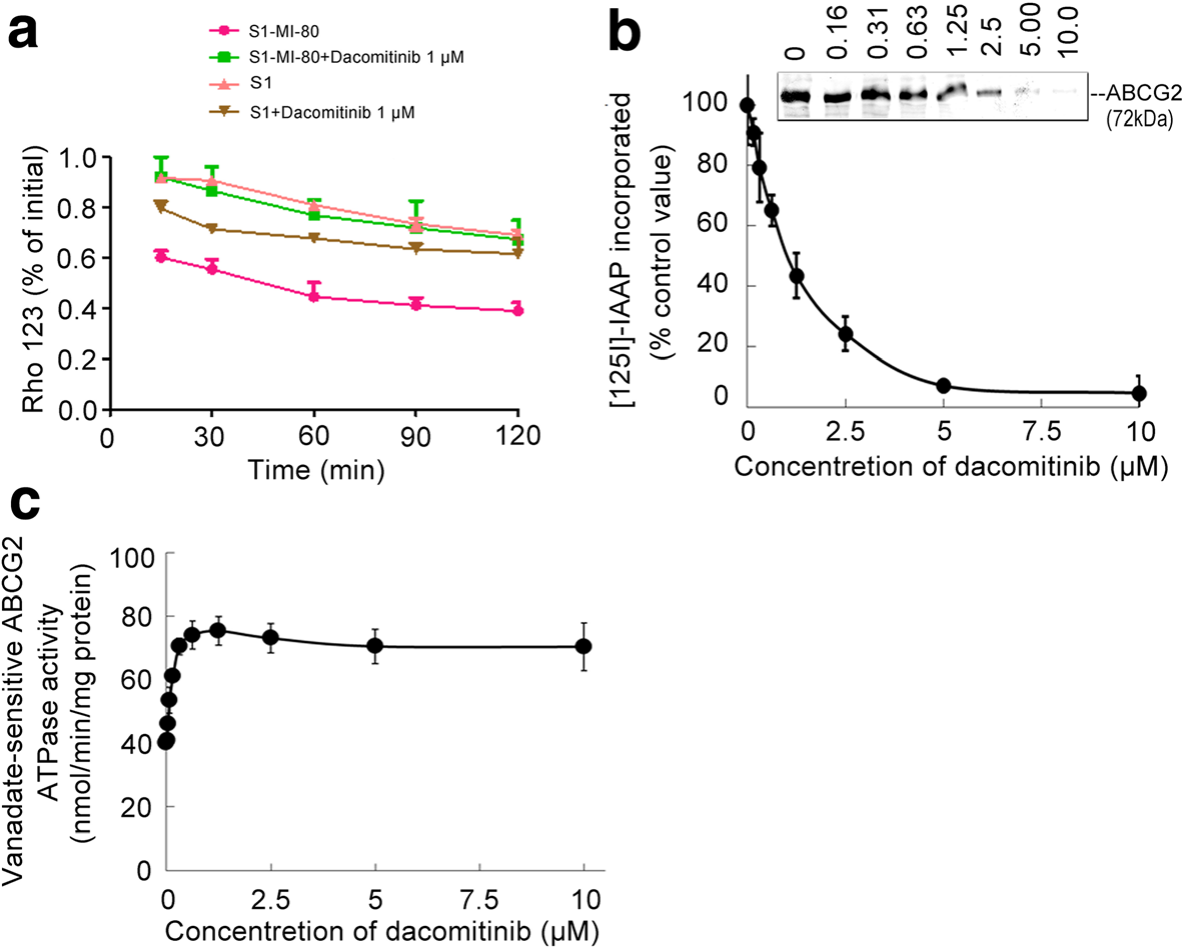
Effect of dacomitinib on the efflux of Rho 123, the ATPase activity and the[^125^I]-IAAP photoaffinity labeling of ABCG2. Time course of Rho 123 efflux was measured in S1 and S1-80-MI cells, with or without 1 μM dacomitinib (**a**). dacomitinib competed for photolabeling of ABCG2 by [^125^I]-IAAP (**b**). Crude membranes from MCF7/FLV1000 cells overexpressing ABCG2 were incubated with [^125^I]-IAAP and a range of different concentration (0–10 μM) of dacomitinib. The samples were then cross-linked by UV illumination, subjected to SDS-PAGE, and analyzed as described in Materials and Methods. A representative autoradiogram from three independent experiments was shown. The relative amount of [^125^I]-IAAP incorporated was plotted against the concentration of dacomitinib used in the competition. 100% incorporation refered to the absence of dacomitinib. Effect of dacomitinib on ABCG2 ATPase activity (**c**). The vanadate-sensitive ABCG2 ATPase activity in the presence of the indicated concentrations of dacomitinib was evaluated. The mean and standard error values from three independent experiments were shown

Since dacomitinib was found to increase intracellular accumulation of DOX and Rho 123 in ABCG2-overexpressing cells, drug efflux assays were performed to further confirm whether it was due to inhibition of substrate drug efflux. We monitored the efflux of Rho 123 at 15, 30, 60 and 120 min after an initial drug accumulation with or without dacomitinib. The efflux of Rho 123 from ABCG2-overexpressing cells S1-MI-80 was significantly higher than that of S1 cells (Fig. 4a). The incubation of dacomitinib at 1 μM could significantly increase the intracellular Rho 123 retention in S1-MI-80 cells. The increase in Rho 123 retention was remarkably more pronounced than that in the parental S1 cells. These results suggested that dacomitinib increased intracellular retention of Rho 123 by inhibiting ABCG2-mediated efflux activity specifically in S1-MI-80 cells.

### Dacomitininb inhibited the [^125^I]-IAAP photoaffinity labeling of ABCG2

While the substrate binding site of ABCG2 could be photo-labeled by [^125^I]-IAAP, transporter substrates were known to compete for the [^125^I]-IAAP labeling. To further ascertain the interaction of dacomitinib with the substrate binding sites of ABCG2, we examined the photo-labeling of ABCG2 with [^125^I]-IAAP by incubating membrane vesicles in the presence of a range of different concentrations of dacomitinib. As showed in Fig. 4b, dacomitinib was found to strongly inhibit the photoaffinity labeling of ABCG2 with [^125^I]-IAAP in a concentration-dependent manner. The result suggested that dacomitinib might compete with ABCG2 substrates to interact with the transporter substrate binding sites, thereby leading to intracellular accumulation of other ABCG2 substrate drugs.

### Dacomitinib stimulated the ATPase activity of ABCG2

The drug-efflux function of ABCG2 was associated with ATP hydrolysis, which was stimulated in the presence of the transporter substrates. To understand whether dacomitinib affected the ATPase activity of ABCG2, we measured the vanadate-sensitive ATPase activity of ABCG2 in the presence of a range of different concentrations of dacomitinib. Dacomitinib was found to stimulate the ATPase activity of ABCG2 in a concentration-dependent manner. A plateau at around 75 nmol/min/mg protein was reached at 1.25 μM dacomitinib (Fig. 4c). The result suggested that dacomitinib might be a substrate of ABCG2.

### Dacomitinib did not alter the expression level of ABCG2

**Fig. 5:**
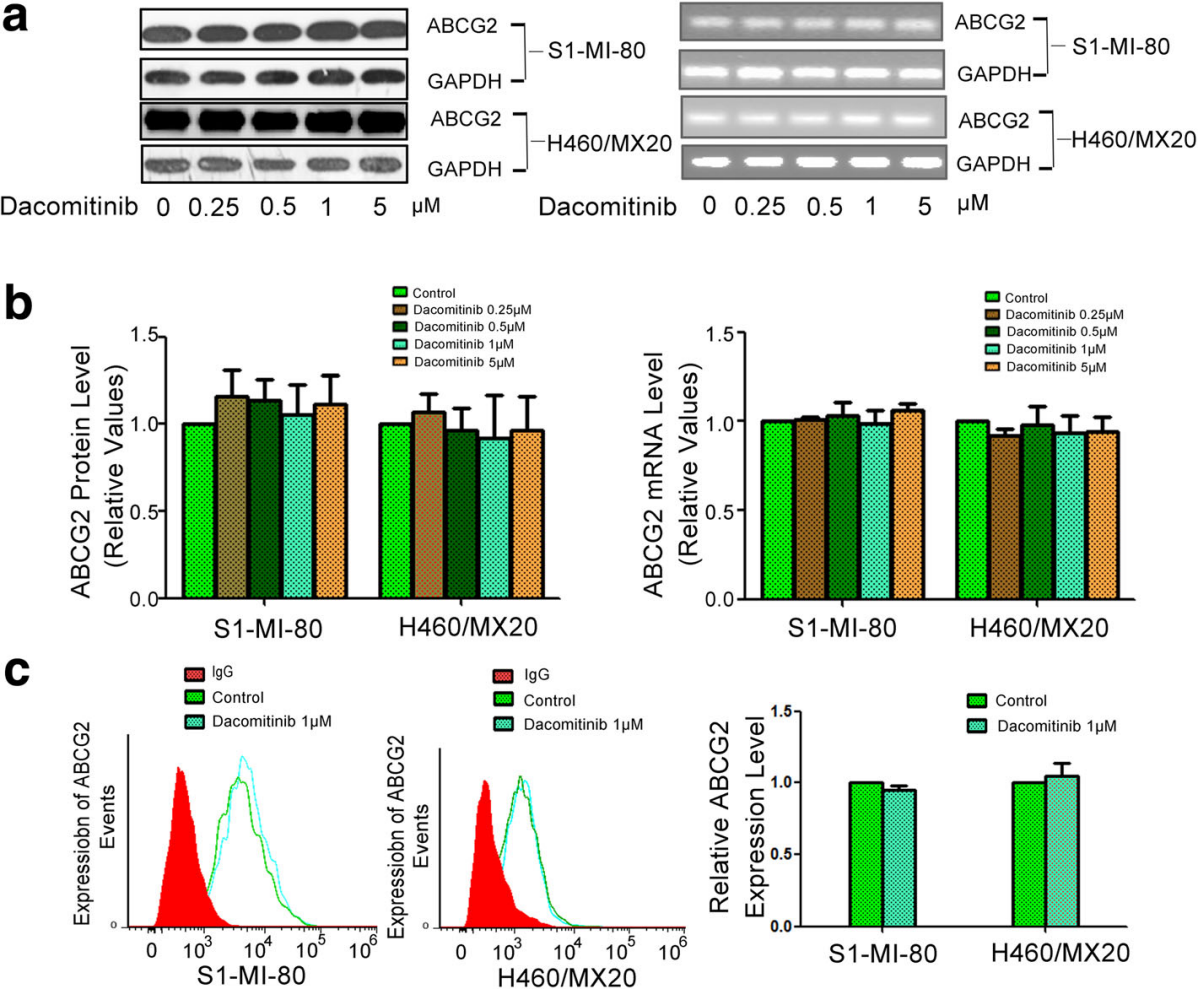
Effect of dacomitinib on the expression of ABCG2 in MDR cells. The protein level of ABCG2 on MDR cells after 0, 0.25, 0.5, 1 and 5 μM dacomitinib stimulation for 48h were measured by western blot analysis, and mRNA level were measured by PCR (GAPDH as loading control) (**a**). Dacomitinib did not alter the mRNA and protein levels. Real time-PCR was further applied to confirm the mRNA expression levels in MDR cells in the presence or absence of dacomitinib (**b**). The cell surface expression of ABCG2 were measured by flow cytometry in the presence or absence of dacomitinib in MDR cells and their parental cells (**c**). All these experiments were repeated at least three times, and representative images and densitometry results were shown in each panel. Columns, means of triplicate determinations

The reversal of ABCG2-mediated MDR could be achieved by either antagonizing the function of efflux pump or decreasing the expression levels of the ABCG2 [34]. Therefore, expression level of ABCG2 was also monitored in the presence or absence of dacomitinib. We measured the expression of ABCG2 at protein and mRNA level in H460/MX20 and S1-MI-80 cells after treated with a range of varying concentrations of dacomitinib. No significant difference in ABCG2 expression at both mRNA and protein level was observed (Fig. 5a, b). Thus, the reversal of ABCG2-­mediated drug resistance was unlikely caused by the reduction of ABCG2 expression.

### Dacomitinib did not significantly alter the cell surface localization of ABCG2

To further evaluate whether dacomitinib could influence the cellular localization of ABCG2, we analyzed the cell surface expression of ABCG2 in the presence or absence of 1 μM dacomitinib in ABCG2-overexpressing drug resistant cells. The surface expression of ABCG2 was not significantly altered in S1-MI-80 and H460/MX20 cells with or without 1 μM dacomitinib. The results indicated that the cell surface localization of ABCG2 was not affected by dacomitinib (Fig. 5c).

### Dacomitinib did not block the phosphorylation of AKT and ERK at MDR reversal concentrations

**Fig. 6:**
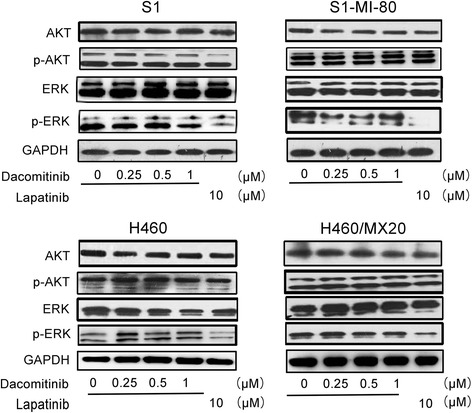
Effect of dacomitinib on AKT, ERK, and their phosphorylations in MDR and the parental drug sensitive cells. H460/MX20, S1-MI-80 and their parental drug sensitive cells were treated with different concentrations of dacomitinib, and 10 μM of lapatinib was used as positive control for the blockage of ERK phosphorylation for 48 h. The total and the phosphorylation protein level of AKT and ERK were detected by western blot (GAPDH as loading control). All these experiments were repeated at least three times

It has been reported that sensitivity of cancer cells to chemotherapeutic agents could be increased by blocking downstream oncogenic signaling of AKT or ERK phosphorylation [32]. Therefore we examined whether the enhancement effect of dacomitinib was related to blockage of AKT and ERK pathway in H460/MX20, S1-MI-80 and their parental cells at MDR reversal concentrations. At the tested MDR reversal concentration (up to 1 μM dacomitinib), AKT and ERK phosphorylation was not affected (Fig. 6). As a control, lapatinib (at 10 μM) known to inhibit ERK phosphorylation was observed. The data suggested that the effect of dacomitinib on reversal of ABCG2-mediated MDR was not associated with the blockade of downstream AKT and ERK signaling.

## Discussion

Inhibition of the drug transport function of ABC transporters is a promising strategy to reverse drug resistance in cancer treatment. To date, enormous efforts have been devoted to the development of ABC transporter inhibitors. However, the application of these ABC transporter inhibitors in clinical practice is limited because of side effects or unexpected pharmacokinetic interactions. We have been studying the use of molecularly targeted TKIs to inhibit ABC transporters in the circumvention of MDR for many years. We found that TKIs could inhibit drug efflux activity of ABC transporters at low concentrations and potentiate anti-cancer effect of chemotherapeutic drugs in cancer cells. Therefore, we speculated that TKIs might be repositioned as inhibitors of ABC transporters to circumvent MDR in the clinic.

Dacomitinib is a promising second generation EGFR TKI exhibiting potent anticancer activity *in vitro*. More importantly, results of phase III clinical trials also revealed that dacomitinib could significantly improve disease PFS, when compared with the first generation EGFR TKI erlotinib [35]. Moreover, PFS benefit was observed in most clinical and molecular subsets, notably KRAS wild-type/EGFR any status, KRAS wild-type/EGFR wild-type, and EGFR mutants [36, 37]. In this study, we investigated the circumvention of ABCG2-mediated MDR by dacomitinib *in vitro* and *in vivo*.

**Fig. 7:**
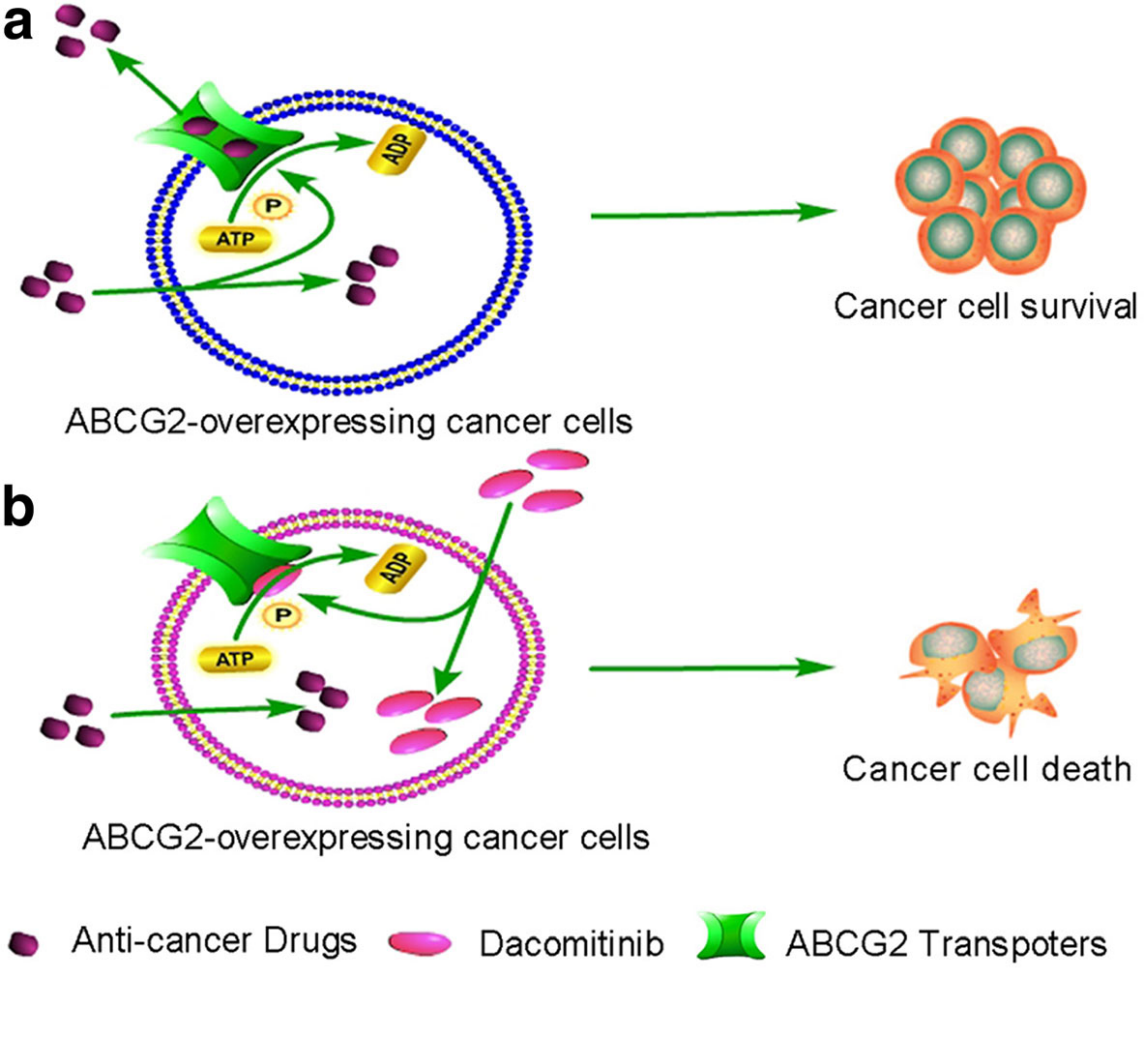
Schematic diagram of dacomitinib reversing MDR**.** In the absence of dacomitinib, ABCG2 transporters utilize energy derived from the hydrolysis of ATP to efflux its substrates agents crossing the membrane (**a**). However, in the presence of dacomitinib, dacomitinib may bind to the ATP binding site of ABCG2, thereby blocking the efflux of the transporter substrates and increasing the intracellular accumulation of the substrate drugs. Therefore, dacomitinib could increase the efficacy of conventional chemotherapeutic drug in ABCG2 overexpressing cancer cells (**b**)

Dacomitinib was first evaluated for MDR reversal in cancer cell models with defined overexpression of ABCG2. While dacomitinib at concentration below 1 μM did not appreciably affect cell proliferation, it was found to significantly potentiate the anticancer activity of other ABCG2 substrate anticancer drugs in two drug-selected ABCG2-overexpressing cancer cell lines (H460/MX20 and S1-MI-80) and two ABCG2 stable transfected HEK293 cells (expressing either the wild type ABCG-482-R2 or the mutant ABCG-482-T7). The MDR reversal was specific because no appreciable effect was observed in the parental cancer cells (H460 and S1) or the backbone vector transfected HEK293/pcDNA3.1 cells. Moreover, dacomitinib did not significantly alter anticancer activity of non-ABCG2 substrates on cancer cells. The data on the wild-type ABCG2-482-R2 and mutant ABCG2-482-T7 is useful because the polymorphism at 482 may change the chemotherapeutic drug selectivity [24]. The promising data *in vitro* was also verified *in vivo* in the H460/MX20 cell xenograft nude mice model. Dacomitinib was also found to significantly enhance the antitumor activity of an ABCG2 substrate drug topotecan in H460/MX20 cell xenografts without increasing toxicity. *In vitro* and *in vivo* data suggests that dacomitinib may be a great candidate of ABCG2 inhibitors, which advocates further investigation of combination chemotherapy of dacomitinib with conventional anticancer drugs in the cancer patients with ABCG2 overexpression. Reversal of ABCG2-mediated MDR may involve either inhibition of the efflux function or reduction in expression of the transporter. To explain this, drug accumulation and efflux activity were measured in ABCG2-overexpressing cancer cells by flow cytometry. The data suggested that dacomitinib inhibited efflux of ABCG2 substrates out of the cells, thereby resulting in an increase of intracellular accumulation of ABCG2 substrates such as Rho123, DOX and Hoechst 33342. Since drug efflux by ABCG2 depends on energy derived from ATP hydrolysis by ATPase, we detected the activities of ABCG2 ATPase in the presence or absence of dacomitinib. Dacomitinib stimulated ATPase activity in a concentration-dependent manner, suggesting that dacomitinib may be a substrate of ABCG2. This is consistent with the competition by dacomitinib for [^125^I]-IAAP photo-affinity labeling of ABCG2. Dacomitinib likely binds to the ATP binding sites of ABCG2. The schematic diagram illustrating the reversal of MDR by dacomitinib was showed in Fig. 7.

ABCG2 expression at both mRNA and protein levels were also evaluated in the resistant ABCG2-overexpressing cancer cells H460/MX20 and S1-MI-80 after treatment with dacomitinib at the MDR reversal concentrations for 48 h. Data from real time PCR and Western blot analysis showed that mRNA and protein expression levels of ABCG2 were not altered by dacomitinib treatment. Therefore, the MDR reversal observed in the study may be mediated mainly by the inhibition of ABCG2 efflux activity by dacomitinib.

Moreover, we found that dacomitinib did not block the phosphorylation of AKT and ERK in H460/MX20 cells, S1-MI-80 cells and their parental cells at MDR reversal concentration of 1 μM. Thus, the potentiation of the cytotoxic effects of anti-cancer drugs by dacomitinib in MDR cells was not related to the inhibition of EGFR or Her-2 receptors and their downstream signaling pathways.

## Conclusions

Dacomitinib may be adopted as a novel chemosensitizer to overcome MDR in ABCG2-overexpressing cancer cells. Further clinical studies are warranted to fully appreciate the beneficial combination of dacomitinib and other conventional anticancer drugs in cancer patients.

## Abbreviations

ABC: Adenosine triphosphate (ATP)-binding cassette
ABCG2: ABC transporter subfamily G member 2
DDP: Cisplatin
DMSO: Dimethyl sulfoxide
DOX: Doxorubicin
FTC: Fumitremorgin C
IAAP: Iodoarylazidoprazosin
MDR: Multidrug resistance
MTT: 3-(4,5-dimethylthiazol-2yl)-2,5-diphenyltetrazoliumbromide
MX: Mitoxantrone
PCR: Polymerase chain reaction
PFS: Progression-free survival
Rho 123: Rhodamine 123
VRP: Verapamil
